# Experimental and Simulation Studies on Hematite Interaction with Na-Metasilicate Pentahydrate

**DOI:** 10.3390/molecules28083629

**Published:** 2023-04-21

**Authors:** Gonzalo R. Quezada, Norman Toro, R. S. Krishna, Subhabrata Mishra, Pedro Robles, Ivan Salazar, Enoque Mathe, Ricardo I. Jeldres

**Affiliations:** 1 Escuela de Ingeniería Química, Facultad de Ingeniería, Universidad del Bío-Bío, Concepción 4081112, Chile; 2 Faculty of Engineering and Architecture, Universidad Arturo Prat, Iquique 1100000, Chile; notoro@unap.cl; 3 Indian Institute of Technology Guwahati, Technology Innovation Hub, Guwahati 781039, India; krishnaskull@gmail.com; 4 CSIR—Institute of Minerals and Materials Technology, Bhubaneswar 750103, India; subhabrata.21@immt.res.in; 5 Academy of Scientific and Innovative Research, Ghaziabad 201002, India; 6 Escuela de Ingeniería Química, Pontificia Universidad Católica de Valparaíso, Valparaíso 2340000, Chile; pedro.robles@pucv.cl; 7 Department of Civil Engineering, Universidad Católica del Norte, Antofagasta 1270709, Chile; isalazar@ucn.cl; 8 Departamento de Ingeniería Química y Procesos de Minerales, Facultad de Ingeniería, Universidad de Antofagasta, Antofagasta 1240000, Chile; enoque.dinis.mathe@ua.cl (E.M.); ricardo.jeldres@uantof.cl (R.I.J.)

**Keywords:** hematite, sodium metasilicate, rheology, molecular dynamics, interaction mechanisms

## Abstract

Iron ore is a fundamental pillar in construction globally, however, its process is highly polluting and deposits are becoming less concentrated, making reusing or reprocessing its sources a sustainable solution to the current industry. A rheological analysis was performed to understand the effect of sodium metasilicate on the flow curves of concentrated pulps. The study was carried out in an Anton Paar MCR 102 rheometer, showing that, in a wide range of dosages, the reagent can reduce the yield stress of the slurries, which would result in lower energy costs for transporting the pulps by pumping. To understand the behavior observed experimentally, computational simulation has been used by means of quantum calculations to represent the metasilicate molecule and the molecular dynamics to study the adsorption of metasilicate on the hematite surface. It has been possible to obtain that the adsorption is stable on the surface of hematite, where increasing the concentration of metasilicate increases its adsorption on the surface. The adsorption could be modeled by the Slips model where there is a delay in adsorption at low concentrations and then a saturated value is reached. It was found that metasilicate requires the presence of sodium ions to be adsorbed on the surface by means of a cation bridge-type interaction. It is also possible to identify that it is absorbed by means of hydrogen bridges, but to a lesser extent than the cation bridge. Finally, it is observed that the presence of metasilicate adsorbed on the surface modifies the net surface charge, increasing it and, thus, generating the effect of dispersion of hematite particles which experimentally is observed as a decrease in rheology.

## 1. Introduction

Iron and steel demand has increased significantly, and the world crude steel production accounted for more than 1900 MTPA by 2021–2022 [1]. The production of iron and steel is directly dependent on iron ore mining, which is the primary raw material source of iron and steel production [1]. Additionally, iron and steel scraps are secondary raw material resources. However, due to the depletion in the grade of iron ores, researchers and industries are looking for the sustainable utilization of medium, low, and lean grades of iron ores, including tailings and slimes [2,3,4,5]. During the beneficiation of these medium and low-grade iron ores, a considerable amount of water is used to carry out unit operations, such as wet high-intensity magnetic separation (WHIMS), Desliming, etc. Dewatering the final concentrate and tailing is vital to recovering the water for its reutilization concerning environmental constraints and cost economics [6,7,8].

Moreover, the direct discharge of tailing/slime slurry into rivers and ponds harms the nearby living bodies as it contains many heavy metals, which must be removed from the wastewater before being discarded [9]. Gravity thickening often represents the most practical choice to achieve high throughput with the desired performance, even though filtration and centrifugation are frequently utilized for solid-liquid separation [10]. Additionally, flocculation-magnetic separation is more efficient and cost-economical for separating finely weak magnetic iron ore particles [11]. Thickeners are extensively used to retrieve water from concentrate and tailing fractions, which are being used for different plant activities [7,8,12]. Usually, the thickeners enrich the solid concentrations of the slurry samples being collected from the beneficiation stages [11,12]. These concentrated slurries must be pumped from the bottom of the thickener to the tailings storage facilities. Transport energy consumption can be high for concentrated pulps when the rheological parameters increase. In this context, process engineers are constantly looking for alternatives to reduce the rheological parameters of concentrated pulps, including pH modifications, pulp density reduction, shear-thinning strategies, or new chemical reagents that increase particle dispersion.

The slurry settles inside the thickeners by sedimentation principle, where hindered settling occurs [13]. However, settling these slurry samples depends on many parameters such as pH, particle size, density, etc. The settling is affected due to ultrafine particles and a higher slime content in the case of iron ores which may require further chemical treatments to acquire the desired settling rate [8]. Selective flocculation is needed, which agglomerates these ultrafine particles called flocs; hence, the flocs settle down using gravity [8]. Polymeric flocculants are widely used in mineral processing industries for dewatering applications that are available in the form of natural and synthetic. The natural ones are biodegradable and environmentally friendly, however, they function at a higher dosage that is not viable for industrial applications [11,14].

Contrary to this, synthetic polymers are efficacious even at low dosages [13]. Polyacrylamides (PAMs) and their derivatives, such as partially hydrolyzed polyacrylamide (HPAM), Amphoteric PAM (aPAM), and Cationic PAM (cPAM), are widely used synthetic polymers in various mineral processing units [8,9,15,16,17,18]. Many studies were carried out using PAM-based flocculants to understand the interfacial chemistry of the hematite-bearing low-grade iron ore [17,18]. Additionally, the impact of the anionic and non-ionic PAM flocculants on the settling behavior of iron ore, their interfacial chemistry, rheology, and the dewatering model was explored experimentally [19]. Moreover, sedimentation of hematite using anionic PAM (APAM) flocculants depends on various factors such as regulated time, pH, and the presence of other hydroxyl complex ions like Fe(OH)^2+^ and Fe(OH)^2+^ [20]. Further, the presence of Fe^3+^ ions in the APAM decreases its degradation capability, resulting in a reduced flocculating ability [20]. In addition to this, the use of sodium metasilicate for the flocculation of hematite has been reported by various researchers [12,21,22,23,24]. Implementing sodium metasilicate increases the settling rate, however, it depends on the pH of the slurry [12]. Furthermore, settling iron ore by adopting PAM, HPAM, and sodium metasilicate as a dispersant has an improved settling performance due to the increased adsorption rate [22].

The computer modeling and the simulation study of the sedimentation and selective flocculation has been widely adopted, which enables researchers to actualize the process on an absolute scale [23]. The molecular dynamics of additives and dispersants can be visualized under the computer simulation environment, which helps to comprehend the process on a micron scale between them [24]. Many articles have reported the simulation study by adopting a molecular dynamics approach to visualize the flocculation mechanism under a computer environment [25,26,27,28,29]. Before this, the authors used molecular dynamics to investigate the flocculation behavior of binder polymers PAM, HPAM, and sodium silicate as dispersants [22]. The study revealed that the presence of sodium metasilicates promotes the adsorption process. However, due to the presence of salts, the adsorption is affected during the usage of PAM. In contrast, HPAM performs better as the salt promotes the formation of cationic bridges and attracts the hematite surface [22]. Another work performed an experimental and atomistic simulation by introducing a selective flocculant (St-Meth-Co-PAM) where the starch methacrylate (St-Meth) has been dispatched to the PAM by the free radical polymerization process. The synthesized flocculant, i.e., St-Meth-Co-PAM, exhibited exceptionally well in separation efficiency [30]. Additionally, the selective flocculant binds hematite particles more effectively than starches, as seen in the atomistic simulation study [30].

The presence of metasilicate in the sedimentation process of iron ore helps remove the multivalent cations, such as calcium and magnesium, that cause coagulation [14]. The present study emphasized the metasilicate-assisted settling of hematite, which appeared very few in the literature. Hence, the current work aims to see the effect of sodium metasilicate on the mineral hematite under a rheological characterization that may improve the pumping conditions for the slurries. A study of molecular absorption by molecular dynamics is performed to understand the adsorption mechanisms of the reagents on the mineral surface.

## 2. Results

### 2.1. Rheology

**Figure 1:**
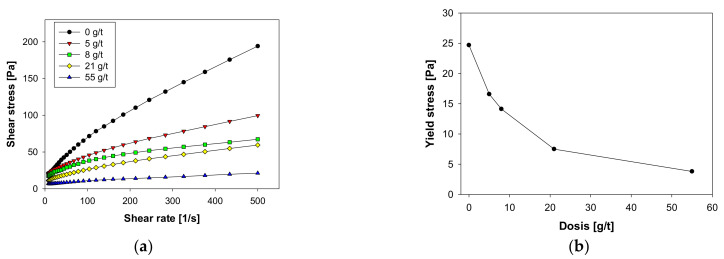
Impact of sodium metasilicate on the rheological behavior of Hematite suspensions in 0.01 M NaCl. (**a**) Full rheogram, (**b**) Yield stress.

Figure 1 shows the rheological behavior of the hematite suspensions, analyzing the impact of sodium metasilicate as a rheological modifier. Figure 1a shows the rheograms obtained in the 0–500 s^−1^ range, noting that in all cases, a shear thinning behavior appears with a yield stress that must be overcome for the pulp to begin to flow. The yield stress can be controlled by adding the dispersing reagent, sodium metasilicate, which significantly reduced its value (Figure 1b). The operational implications of this phenomenon are of particular importance for the mining industry when it refers to the transport of concentrated slurries, which is usually carried out by pumping, whose electrical consumption depends directly on the rheological parameters. In this context, a decrease in yield stress leads to economic and energy savings after applying low doses of sodium metasilicate. In this context, the anionic reagent adheres to the mineral surface by the adsorption mechanisms that are analyzed in the subsequent sections. This confers a greater anionic charge to the hematite surface, generating an electrostatic dispersion of the particles.

### 2.2. Quantum Calculations

**Figure 2:**
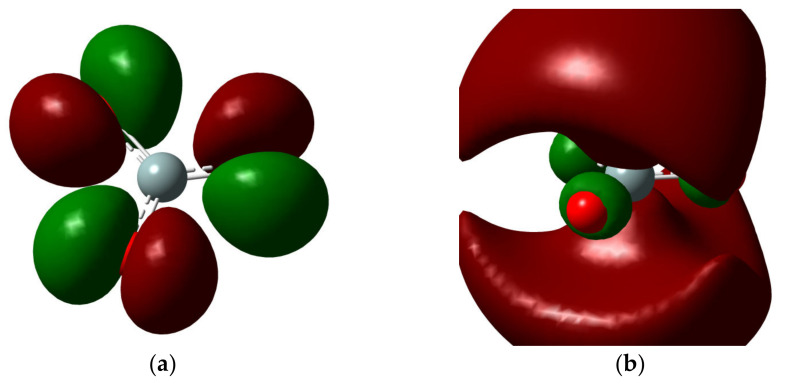
Quantum calculations for SiO_3_ molecule. (**a**) HOMO state and (**b**) LUMO state.

In the case of the results of the quantum calculations, optimization and frequency calculation was used to obtain the calculation of the partial charges in a stable way. In this case, the base structure of the silicate was used, which determined its LUMO and HOMO formations to determine its reactivity. Figure 2 shows how electronic clouds are in their state. When observed quantitatively, it is observed that the HOMO state presents the greatest reactivity in its oxygen groups. However, we see that in the LUMO state, which is necessary to effect chemical reactions, it is observed that electronic clouds have a high population above the silica group. This indicates that this molecule can react with a molecule, for example water, and form the silicate H_2_SiO_4_.

**Figure 3:**
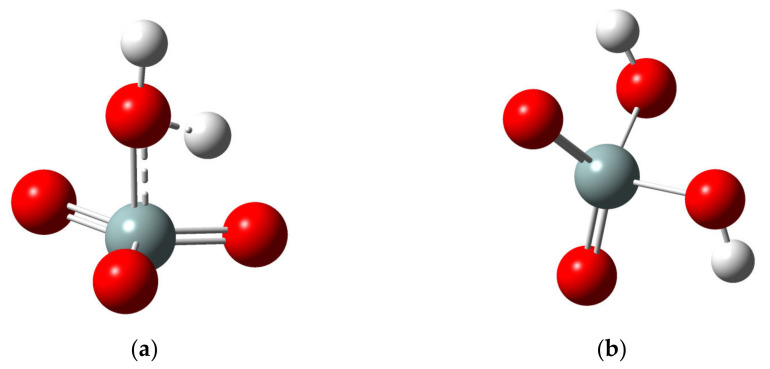
Quantum calculations for reaction of H_2_O with SiO_3_ molecule. (**a**) Initial configuration (**b**) final configuration.

Then a minimization was made by placing the SiO_3_ molecule in the presence of a water molecule. This is observed, in Figure 3, where the water molecule has been placed in the area where the SiO_3_ molecule is more reactive. Then when performing the optimization, the final molecule is shown in Figure 3b. We can observe that part of the water molecule (OH group) is bonded directly on the silica atom, while a hydrogen atom of the water molecule is bonded with the oxygen of the SiO_3_ molecule.

Finally, the RED program is used to calculate partial loads. In this case, it is then obtained that the silicon atom has a partial charge of +1.2517e, the oxygen with a double bond to silicon has a partial charge of −1.112e, the oxygen that is attached to the hydrogen and the silicon atom has a partial charge of −0.86265e, and the hydrogen atom has a partial charge of +0.3488e. The force field for the bonds, angles, and dihedral was also scanned by means of quantum calculations, but because it was like the CVFF force field, it was preferred to use this force field.

### 2.3. Adsorption Simulation

**Figure 4:**
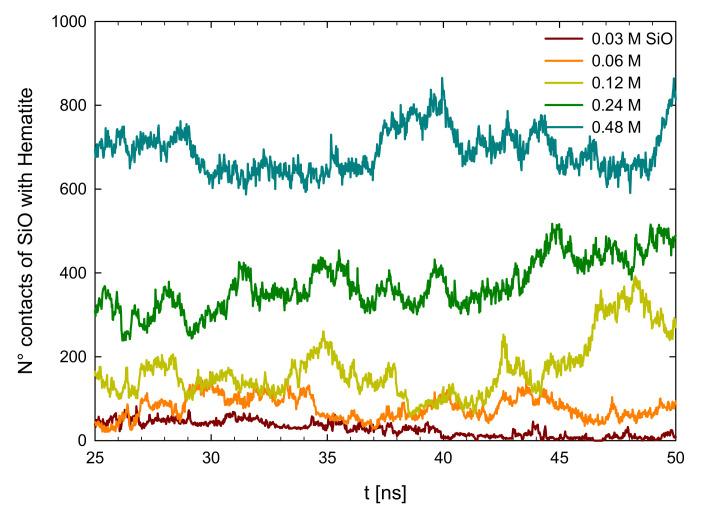
The number of contacts between metasilicate and hematite surface at different metasilicate concentrations.

The adsorption simulations were carried out at different sodium metasilicate concentrations. Figure 4, shows the evolution of the contacts between the surface with the molecules of metasilicate over time, mainly measuring the pairs of atoms between the surface atoms with the atoms of the metasilicate. It can be observed that as the concentration of metasilicate increases, the number of molecules interacting with the surface increases. It is observed, for example, that with 0.03 M of metasilicate, we have a number of contacts of ~50. Then at 0.06 M, this increases to about 100. Then at 0.12 M an amount between 180 is observed and there is a small rise at the end, but this goes down, so clearly the adsorption reaches a stable adsorption. Then for the highest concentrations of 0.24, we have between 300 to 400 contacts, and finally, for 0.48 M, we see several contacts of 700. We can see here that there is a strong affinity between the metasilicate molecules and the hematite surfaces.

**Figure 5:**
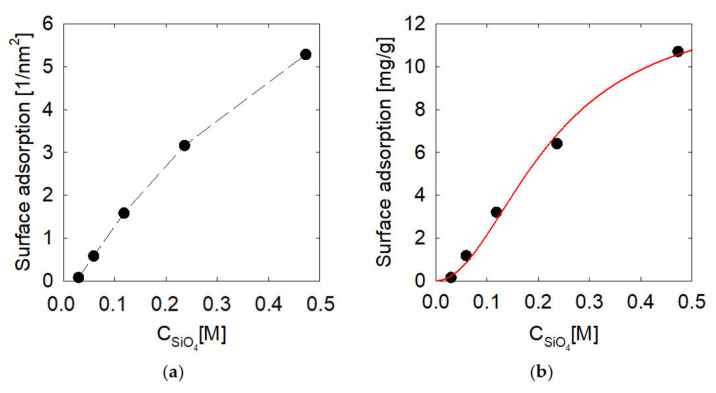
Mean surface adsorption of metasilicate on hematite (001) surface at different metasilicate concentrations. (**a**) in silicate molecules by nm^−2^ and (**b**) in mg of silicate by g of hematite with the fitted sips model.

Then it is possible to transform the data from the contact numbers to surface adsorption using Equation (2). This then generates an intensive magnitude of adsorption, the results in both units of nm^−2^ and mg/g are in Figure 5. We observe a steady growth of sodium metasilicate adsorption in Figure 5a, within the range of 0 to 0.5 M, where it increases from 0 molecules per nm^2^ to 5.2 molecules per nm^2^. The results have also been reported using the equation in Section 4.3. This is seen in Figure 5b, which increases from 0 mg/g to 11 mg/g. If we fit the Sips model, we have that $q_{m}$ is equal to 12.9 mg/g and that $K_{S}$ is equal to 20.053 (l/mol)^n^, where n is 0.5. This indicates that the equilibrium adsorption of metasilicate is almost obtained on the surface under this model, and therefore, we reach a concentration where more metasilicate could not contribute to the dispersion of the hematite particles.

**Figure 6:**
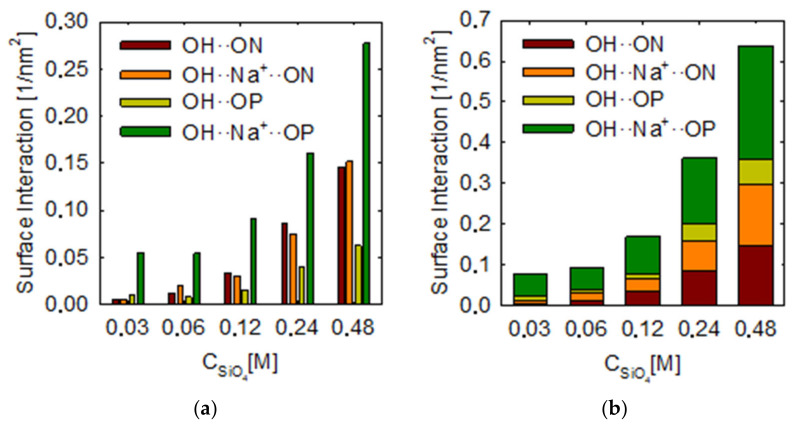
Mean surface interaction of metasilicate on hematite (001) surface at different metasilicate concentrations. (**a**) groups bars and (**b**) stacked bars.

Next, the interactions of the surface with the metasilicate were determined by hydrogen bridging interaction, either OH∙∙ON which represents the interactions between the FeOH group of the surface with the OH of the metasilicate, or OH∙∙∙OP which is the interaction between the FeOH group of the surface with the =O of the metasilicate. Additionally, included are the cationic-type interactions where the Na+ cation binds the bridge, either with OH∙∙Na+∙∙∙ON or OH∙∙∙Na+∙∙∙OP. The results are shown in Figure 6, where Figure 6a shows the separate contribution of all four types of interactions and Figure 6b shows the whole interactions at different metasilicate concentrations. 

In general, it is observed that interactions of all types increase as the metasilicate concentration increases, which is evident because more molecules are interacting with the surface. Analyzing the hydrogen bridge interactions, it is seen that for OH∙∙ON, the values grow from 0.005 to 0.14 nm^−2^.In the case of OH∙∙OP, their values range from 0.01 to 0.06 nm^−2^. The OH∙∙∙Na+∙∙ON cation bridges also show an increase from 0.005 to 0.15, like that observed for OH∙∙∙ON. In the case of OH∙∙Na+∙∙∙OP, it is observed to increase from 0.05 to 0.27 nm^−2^. 

It is observed from these analyses that the highest interaction occurs for OH∙∙∙Na+∙∙∙OP interactions, indicating that the presence of sodium is relevant for higher metasilicate adsorption. Nevertheless, we see that the contribution of OH∙∙Na+∙∙∙ON and OH∙∙ON are also relevant, where the OH of the metasilicate also interacts with the surface, the reason that interactions of OH∙∙∙Na+∙∙∙OP are strong is that, as we see in previous works [31,32], the sodium is adsorbed strongly to an XOO group, in this case, SiOO where both oxygens are the double bond oxygens (O=).

### 2.4. Surface Charge and Sodium Density

**Figure 7:**
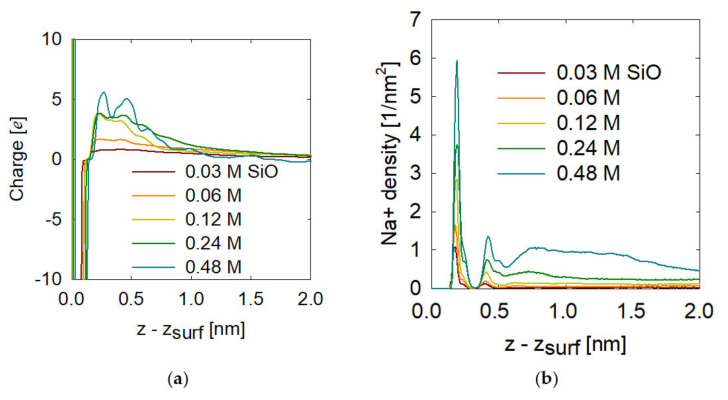
(**a**) Charge and (**b**) Na^+^ density profile for the hematite (001) surface for all the concentrations of metasilicate.

Finally, the effective charge calculations were performed along the z direction starting at the surface in Figure 7a. As a reference, the surface oxygen, FeOH, was taken, which is why at z equal to zero, the charge is negative and then neutralized around 0.12 nm by the hydrogen of the FeOH group. After 0.12 nm above the reference, the charge increases to positive values. We can see from Figure 7a that at low concentrations of metasilicate, the vicinity on the surface has a load of approximately zero, this is within a range between 0 to 2 nm on the surface. If we increase the concentration of metasilicate (we know that it is adsorbed on the surface as shown in Figure 5), we can see that at 0.06, the net load on the surface begins to increase to a value of +1e at 0.25 nm that is then neutralized at greater distances from the surface. If we continue to increase, we see that at 0.48 M, the vicinity on the surface shows an appreciable positive charge, with maximums of +5e. This means that the net surface load increases to a constant value in its vicinity, between 0 to 1 nm. As shown in Figure 6, this phenomenon is not only from the adsorption of metasilicate but also from the adsorption of sodium cations from metasilicate.

Therefore, it is necessary to observe how the adsorption of the sodium cation on the surface was quantified by the density profiles of the sodium cation on the surface shown in Figure 7b. Evidently, when the concentration of metasilicate increases the adsorption of sodium increases, this is a combined effect and both require themselves to be adsorbed as seen in Figure 6. We see that there are two stable positions of sodium adsorption, at 0.25 nm and 0.4 nm, this is consistent with the increases in the net surface charge of Figure 7a.

## 3. Materials and Methods

### 3.1. Materials

**Figure 8:**
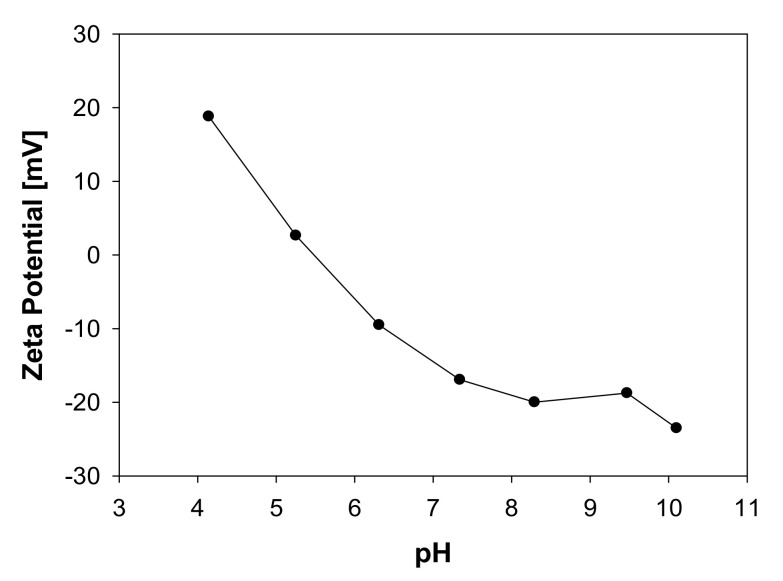
Impact of pH on zeta potential of Hematite particles (-325# mesh) in distilled water (0.01 M NaCl).

The hematite rocks were purchased from Ward’s Science. These underwent a comminution process to achieve a 100% size under 325 mesh. Sodium metasilicate, Na_2_SiO_3_, was purchased from Merck (Darmstadt, Germany). Industrial water was prepared with 0.01 M analytical grade NaCl from Merck (Darmstadt, Germany). Figure 8 shows the particles’ zeta potential as a pH function. It is observed that the isoelectric point is around 5 pH. In contrast, at the natural pH of the pulp (9 pH approx), the particles present a value of −20 mV approx, suggesting that there will be a certain electrostatic repulsion between the particles. The measurements were made in an Anton Paar LiteSizer 500 particle analyzer.

### 3.2. Rheological Tests

Hematite pulp was prepared at natural pH in a vessel at the appropriate solids and reagent concentration as described below. After 1 h of mixing, a 56 mL aliquot was taken for rheological analysis. An Anton Paar MCR 102 rheometer with the RheoCompass Software operated at a controlled rate mode, and a sandblasted #CC39 bob-in-cup configuration (gap 1.5 mm) was used to reduce the wall slip effects. The starting value of the shear rate was preset at 10 s^−1^ with a maximum value of 500 s^−1^. The temperature of the sample was kept constant at 23 °C. The yield stress was calculated by fitting the experimental data to the constitutive equation of the Herschel-Bulkley (see Equation (1)).

(1) $$\tau =\tau_{0}+k{\dot{\gamma }}^{n}$$

where τ is the shear stress, $\dot{\gamma }$ the shear rate, $\tau_{0}$ the yield stress, k the consistency index, and n the flow index.

## 4. Computer Simulation

### 4.1. Force Field

For the mineral slab, the CLAYFF-MOH was used [33,34]. This force field avoids the over-parameterization of crystalline structures, considering mainly nonbonded parameters. The hydroxyl groups on the surface were parameterized to reproduce their behavior correctly. Mainly the bond parameters represent the OH groups by an Morse function and the M-OH angles (where M can be Si, Al, or Mg) by a harmonic function. 

In the case of the silicate ions, quantum calculations were made using the Gaussian 09 software to determine the partial charges using the B3LYP functional with a 6–31 g basis set. In the case of Lennard-Jones, a consistent valence force field (CVFF) parameters were used [35]. For the case of the Na^+^ ions, the Lennard-Jones 12-6 potential was used where the parameters were taken from Li et al. [36]. This work has adjusted parameters for different water models, taken as an objective to adjust both the hydration-free energy (HFE) and the ion oxygens distance (IOD) in terms of the potential 12-6. In this case, the optimized parameters for the IOD were used, where the associated error in the HFE calculations is less than 5%. The SPC/E model [37], constrained with SETTLE [38], was employed for water. In this case, water is described by a three-atom model with a r_OH_ of 0.1 nm and a θ_HOH_ of 109.47. The partial charges are −0.8476e for the oxygen atoms and +0.4238e for hydrogen ones. This water model can reproduce the properties of the liquid phase under normal conditions with good precision [39]. In addition, the parameters of the ions and surfaces have been adjusted based on the SPC/E model.

### 4.2. System Assembly

The dimensions of a hematite slab were used to design the simulation box, where the optimized unit cell of a cross-sectional area of 0.873 × 0.504 nm^2^ was calculated. This work uses 6 × 10 × 3 supercells, giving a total cross-sectional area of 5.24 × 5.04 nm^2^ (26.38 nm^2^), with a thickness of 1.34 nm. This surface was placed in a rectangular prism simulation box with dimensions Lx × Ly × Lz equal to 5.24 × 5.04 × 12.00 nm^3^ and (α, β, γ) equal to (90, 90, 90). Excluding the slab volume, an initial fluid volume was 281 nm^3^, where the solution was placed. Next, the metasilicate molecules were randomly added to the system away from the surface, and five different concentrations were explored, 5, 10, 20, 40, and 80 molecules, which correspond to a concentration of 0.03, 0.06, 0.12, 0.24, and 0.47 M, respectively. Then the Na^+^ ions were added at no less than 0.5 nm to each atom present. Finally, water is added, avoiding the overlap of water molecules with the atoms already located in the simulation box.

### 4.3. Simulation Details

The molecular dynamics simulations were carried out in the Gromacs 2021.3 simulation package [40]. A force minimization stage was initially carried out with the steepest descent method. Next, an NVT simulation at the isothermal condition of 300 K was carried out, with the entire system immobilized except for the water molecules to form the hydration layers. Then an NP_z_AT simulation stage at 300 K and 1 Bar, where the simulation box was relaxed in the z-direction to keep the isobaric condition, was used. Finally, a 50 ns NVT simulation was used to evolve the free system, where the last 25 ns were used to measure the adsorption of metasilicate on hematite. The integration step was 2 fs and the constants of the modified Berendsen thermostats and barostat were 0.1 ps and 2.0 ps, respectively [41]. The cutoff radius for vdw and coulomb contributions was set to 1.2 nm using the Verlet lists [42]. Long-range corrections were accounted for by PME [43]. The results obtained from the MD are in molecules of SiO by nm^2^ of the surface. To obtain the adsorption in mg of SiO by g of hematite is achieved by:

(2) $$q\left[\frac{mg_{sio}}{g_{hem}}\right]=\Gamma \left[\frac{molec_{SiO}}{nm^{2}}\right]\frac{MW_{SiO}\cdot SA_{hem}}{N_{Av}}10^{21}$$

where MW_SiO_ is the molecular weight of the metasilicate (122.06 g/mol), SA_hem_ is the surface area of the hematite (10 m^2^/g, [44]), and N_Av_ is the Avogadro number. The Sips isotherm is used to adjust the results by:

(3) $$q=q_{m}\frac{K_{s}C_{SiO}^{1/n}}{1+K_{s}C_{SiO}^{1/n}}$$

where $q_{m}$ is the adsorption required to form a monolayer, K_S_ is the Sips constant, n is an exponent constant, and C_SiO_ is the sodium silicate concentration.

## 5. Conclusions

This work has conducted experiments on the behavior of solutions of hematite ore in the presence of sodium metasilicate. It has been characterized that the hematite mineral has an isoelectric point close to five so that at neutral or alkaline pH conditions, it has a slight negative charge. Such behavior implies that a hematite pulp generates a considerable rheology and can hinder handling and transport operations. When introducing doses of sodium metasilicate to the suspension, it can be observed that the rheology decreases considerably. On a real scale, it is inferred that the sodium metasilicate acts as a dispersant when adsorbed on the surface of hematite. Clearly, the electrostatic effect plays an important role and the sodium metasilicate provides a more anionic character to the hematite, promoting the dispersion of particles associated with a significant reduction in the yield stress of the suspension.

On the other hand, by counting on the molecular dynamics results, it has been possible to observe, at the atomic scale, the interaction between metasilicate and the hematite surface and obtain a much more detailed view of what is possible to determine at the experimental scale. It was observed that there is an affinity between metasilicate and hematite through interactions that are mainly cation bridges, which is mediated by a sodium ion exchange between the surface oxygens and metasilicate. It is possible to adjust an adsorption model, called the slips model, which implies that there is a delay in adsorption at low concentrations and then increases and reach a saturation value. These results have helped to understand the mechanisms necessary to be able to adsorb the additives on a hematite mineral to modify, in this case, its rheology, and thus improve its transport properties in mining operations. Future work will be based on continuing to modify the surface of the ore depending on the final objective required.

## Data Availability

The data presented in this study are available on request from authors G.R. Quezada and R.I. Jeldres.
